# Profiling whole-tissue metabolic reprogramming during cutaneous poxvirus infection and clearance

**DOI:** 10.1128/jvi.01272-23

**Published:** 2023-11-27

**Authors:** Luxin Pei, Kirsten E. Overdahl, John P. Shannon, Katherine M. Hornick, Alan K. Jarmusch, Heather D. Hickman

**Affiliations:** 1 Laboratory of Clinical Immunology and Microbiology, National Institute of Allergy and Infectious Diseases, National Institutes of Health, Bethesda, Maryland, USA; 2 Metabolomics Core Facility, National Institute of Environmental Health Sciences, National Institutes of Health, Research Triangle Park, North Carolina, USA; 3 Collaborative Bioinformatics Resource, National Institute of Allergy and Infectious Diseases, National Institutes of Health, Bethesda, Maryland, USA; Northwestern University Feinberg School of Medicine, Chicago, Illinois, USA

**Keywords:** poxvirus, metabolism, immune response

## Abstract

Vaccinia virus (VACV) infection induces prominent changes in host cell metabolism. Little is known about the global metabolic reprogramming that takes place in whole tissue during viral infection. Here, we performed a longitudinal metabolomics study in VACV-infected mouse skin to investigate metabolic changes in the tissue during infection. We assessed metabolites in homogenized skin of the ear pinnae over time in the presence or absence of antigen-specific T cells using untargeted mass spectrometry. VACV infection induced several significant metabolic changes in the tissue, including in the levels of nucleic acid metabolites (reflecting the impact of viral replication on the skin metabolome). Furthermore, monocyte- and antiviral T cell-produced metabolites, such as itaconic acid and glutamine, were significantly increased following infection, highlighting the immune response’s contribution to the global skin metabolome. Additional RNA-Seq of infected skin tissue recapitulated metabolic changes identified by metabolomics analysis. Overall, our study reveals the metabolic balance of viral replication and the antiviral immune response in the skin, elucidating metabolic pathways that could contribute to cutaneous poxvirus control *in vivo*.

**IMPORTANCE:**

Human poxvirus infections have caused significant public health burdens both historically and recently during the unprecedented global Mpox virus outbreak. Although vaccinia virus (VACV) infection of mice is a commonly used model to explore the anti-poxvirus immune response, little is known about the metabolic changes that occur *in vivo* during infection. We hypothesized that the metabolome of VACV-infected skin would reflect the increased energetic requirements of both virus-infected cells and immune cells recruited to sites of infection. Therefore, we profiled whole VACV-infected skin using untargeted mass spectrometry to define the metabolome during infection, complementing these experiments with flow cytometry and transcriptomics. We identified specific metabolites, including nucleotides, itaconic acid, and glutamine, that were differentially expressed during VACV infection. Together, this study offers insight into both virus-specific and immune-mediated metabolic pathways that could contribute to the clearance of cutaneous poxvirus infection.

## INTRODUCTION

Poxviruses have posed a continual threat to human health for centuries. Smallpox, caused by the poxvirus variola virus, was endemic to most of the world even as late as the middle 1900s (1). Case fatalities after infection with variola major were high (around 20%) and individuals that survived often dealt with permanent scarring (1). Edward Jenner famously discovered that the epicutaneous inoculation of a related poxvirus, cowpox, into human skin provided cross-protective immunity against smallpox infection (2, 3). A global public vaccination campaign led to the declaration of the complete eradication of smallpox in 1980. However, sporadic poxvirus outbreaks still occur. Most recently, mpox (MPXV) was introduced into humans and spread rapidly through person-to-person contact, resulting in a global public health emergency (4). The MPXV outbreak highlighted gaps in our knowledge of poxvirus pathogenesis since the eradication of smallpox. Most human poxvirus infections occurred before the advent of sophisticated immunological and metabolic profiling methods. Thus, we still have much to learn about poxvirus immune control and metabolic regulation in the tissue environment.

Viruses induce metabolic alterations to infected host cells. Most virus-induced metabolic changes have been defined using reductionist systems including the *in vitro* infection of cultured cells. The overall metabolic changes induced at the tissue level during viral infection or how immune-mediated viral clearance further perturbs or resolves metabolic deviations from uninfected skin warrants continued investigation. Nonetheless, several unique host cell metabolic requirements for vaccinia virus (VACV) replication *in vitro* have been previously detailed (5, 6). Viral replication consumes host cell macromolecules such as nucleotides, phospholipids, and amino acids to synthesize new viral genomes and assemble progeny virions. *De novo* fatty acid synthesis is needed for VACV viral replication *in vitro*, as chemical antagonists of fatty acid synthesis dramatically impair virion production (5). Additionally, a metabolomics screen of VACV-infected human fibroblasts identified changes in glutamine levels, suggesting that viral replication is dependent on glutamine metabolism (6). In further support, VACV encodes a protein (C16) that enhances glutamine metabolism, which is thought to increase the synthesis of metabolic precursors needed for viral replication (7). Studies have also demonstrated that VACV induces a pseudo-hypoxic state in the host and can inhibit host DNA, mRNA, and protein synthesis to favor viral replication utilizing cellular processing machineries (8, 9). Thus, VACV alters host cellular metabolism in numerous ways to promote successful viral replication.

In addition to metabolic changes occurring in virus-infected cells, viral infection also modifies the tissue’s cellular composition. The rapid recruitment of innate and adaptive cells into the tissue following infection significantly increases metabolic demands. Infected tissues must carefully balance the metabolic requirements of the epithelium along with a robust cellular immune infiltrate to promote viral clearance, limit immune-mediated damage, and heal wounded tissues. Here, we investigated the metabolic changes induced in the skin during VACV infection using a previously established mouse infection model (10, 11). In this model, VACV is inoculated into the mouse ear skin, where the virus replicates productively in epidermal keratinocytes (10). In wild-type mice, skin infection remains localized and is controlled by a robust cell-mediated immune response in the tissue that includes both monocytes and cytotoxic CD8^+^ T cells (10, 11). Monocytes are recruited to the skin after infection and a portion of monocytes become VACV infected. Antigen (Ag)-specific CD8^+^ T cells kill virus-infected monocytes to help limit infection (10). In wild-type animals, viral titers also reveal viral replication in the skin is followed by the gradual clearance of cutaneous VACV infection (10).

Due to their importance in antiviral and anti-cancer immunity, CD8^+^ T cell metabolism has been extensively investigated in the past decade. Cytotoxic T cells have unique energy requirements because of their rapid division, migration, and effector activity. As the overall energy demand increases in response to inflammation and Ag recognition, activated T cells engage in aerobic glycolysis, increase mitochondrial function, and alter mitochondrial morphology (12–14). Besides increased glucose uptake, amino acids and lipids are crucial metabolites for T cell differentiation and function (15–17). Glutamine, the most abundant nonessential amino acid in serum, has been implicated in several aspects of CD8^+^ T cell function and can be metabolized through glutaminolysis to fuel the tricarboxylic acid cycle (18). Furthermore, inhibition of glutamine metabolism reduces T cell production of inflammatory cytokines including interferon-gamma (IFN-γ) and interleukin-17 (IL-17) (19). Host defense against cytomegalovirus and tuberculosis infection depends on glutamine metabolism (19, 20), though an *in vivo* role during the anti-VACV host response has not yet been documented.

In addition to the metabolic requirements of cytotoxic CD8^+^ T cells, macrophages and monocytes also engage in specific metabolic reprogramming to support function. Lipopolysaccharide (LPS)-activated macrophages produce large quantities of itaconate, and this metabolite has been identified in *Mycobacterium tuberculosis*-infected mouse lungs (21–23). In the context of viral infection, mice lacking itaconate-metabolizing enzyme present with increased Zika virus replication and decreased survival (24). Thus, the resolution of infection can be critically dependent upon leukocyte-produced metabolites.

The development of untargeted metabolomics analyses through mass spectrometry affords an unprecedented opportunity to perform a global survey of distinct, virus-induced metabolic changes in whole tissues. Here, we performed whole-tissue metabolomics of the skin to characterize the metabolic signature of T cell-mediated clearance of VACV infection. We identified numerous metabolic changes induced by VACV infection that were independent of T cells, along with changes likely associated with Ag-specific T cell-mediated viral clearance. Concurrently, we performed whole-tissue transcriptomics to better understand the broad metabolic pathways that were significantly altered at the mRNA level. Together, our analyses of the whole-tissue metabolome and transcriptome post-VACV infection provide a deconstructed analysis of the metabolic reprogramming in a virus-infected tissue undergoing immune control.

## MATERIALS AND METHODS

### Mice

*Rag1^-/-^* (Taconic stock number 146) and C57BL/6-Tg(TcraTcrb)1100Mjb (OT-I) T cell receptor (TCR) transgenic mice (Taconic stock number 175) were obtained from the Taconic Farms and National Institute of Allergy and Infectious Diseases (NIAID) Intramural Research Repository. Stock Tg (CAG-DsRed*MST) 1Nagy/J mice (Jackson Laboratory stock #5441) were crossed with OT-I TCR transgenic mice and bred to homozygosity to create dsRed OT-I mice. Six- to 12-week-old female mice were used in all experiments. All animals were housed under controlled conditions of humidity, temperature, and light (12-hour light/12-hour dark cycles), specific pathogen-free conditions (including surveillance and negativity for murine norovirus, mouse parvovirus, and mouse hepatitis virus). Standard rodent chow and water supplied were available *ad libitum*.

### Viruses and infections

Mice were anesthetized using isoflurane with 4% induction and 2% oxygen flow for 3 minutes. Mice were then removed from the isoflurane chamber and epicutaneously infected in the ear pinnae by five gentle pokes with a bifurcated needle dipped in VACV (with working titers of ~2×10^8^ pfu/mL) as previously described (10). VACV-NP-S-eGFP contains a nuclear localizing protein (influenza virus nucleoprotein, NP), the cognate Ag recognized by OT-I CD8^+^ T cells (SIINFEKL, S), and enhanced green fluorescent protein (eGFP). VACV-NP-eGFP is an identical virus lacking SIINFEKL. For these studies, one ear was infected with VACV-NP-S-eGFP (NPS) and the contralateral ear with VACV-NP-eGFP (NPE) as previously described (11).

### Adoptive transfers

Ag-specific CD8^+^ T cells were purified from the lymph nodes and spleen of female dsRed OT-I mice using an EasySep mouse CD8^+^ T cell negative selection kit (StemCell). After purification, cells were tested for purity and activation by flow cytometry to achieve at least 85% purity with greater than 98% CD69^-^ population (CD69 APC clone: H1.2F3, Biolegend 104518). 1 × 10^4^ purified dsRed OT-I CD8^+^ T cells were transferred intravenously into *Rag1^-/-^* recipients 1 day prior to infection for flow cytometry and metabolomics experiments. 2.5 × 10^5^ dsRed OT-I CD8^+^ T cells were transferred for RNA-Seq analysis.

### Flow cytometry

Ear pinnae were harvested after euthanasia at each study time point post infection. Single-cell suspensions of ears were generated by enzymatic digestion of finely chopped ears in collagenase I (4,000 U/mL) for 1 hour at 37°C followed by filtration through a 70 µM nylon cell strainer. Cells were then stained with fluorochrome-conjugated antibodies of CD45 PE-Cy7 (clone: 30-F11, Biolegend 103114), CD8b PerCP-eFluor710 (clone: eBioH35-17.2, ThermoFisher 46-0083-82), CD11b AF700 (clone: M1/70, ThermoFisher 56-0112-82), Gr-1 AF647 (clone: RB6-8C5, Biolegend 108418), and live-dead Zombie Aqua (Biolegend 423102) on ice for 30 minutes. After washing, cells were fixed with 3.2% paraformaldehyde (PFA) for 15 minutes at room temperature. Data were acquired on a 5 laser 16UV-16V-14B-10YG-8R Aurora spectral flow cytometer (Cytek) and analyzed using FlowJo 10.8.0 software (BD). Gating strategy is shown in Fig. S1.

### Viral titers via plaque assay

Ears were harvested at the indicated day post-infection (dpi), then fine chopped and digested by collagenase I for 1 hour at 37°C. Digested tissue suspensions were collected after vigorous pipetting. Samples were kept frozen and processed in a single batch for plaque assay where they were freeze-thawed three times, serially diluted, and plated on 143b thymidine kinase deficient (TK-) cells. TK- cells were incubated for 48 hours, then stained with crystal violet for plaque counting.

### Metabolomics

Ears (*n* = 3 per group) were harvested and weighed on days 5, 6, 7, 8, and 10 post-infection and snap frozen at −80°C. Frozen tissues from all time points were homogenized in a single batch using metal bead homogenization tubes (MP Biomedicals) with high performance liquid chromatography (HPLC)-grade water normalized to tissue weight (50 mg tissue:1 mL H_2_O). After homogenization, 800 µL pre-chilled HPLC-grade methanol was added to 200 µL of tissue homogenate for methanol extraction. Samples were placed in a −20°C freezer to facilitate protein precipitation. The methanolic extracts were centrifuged at 14,000 rcf at 4°C for 10 min and the supernatant was vacuum concentrated using a SpeedVac.

Untargeted metabolomics was then performed using ultra-high performance liquid chromatography–high-resolution tandem mass spectrometry (UHPLC-HRMS/MS). Detailed methodology is provided in Supplemental Methods. All chemicals identified and referenced annotations are listed in Tables S1 and S2. Additional information on specific chemicals referenced in main figures are provided in Table S3. In brief, samples were analyzed using an ultra-high performance liquid chromatograph (Vanquish, Thermo Scientific) coupled to a high-resolution mass spectrometer (Orbitrap Fusion Tribrid, Thermo Scientific). LC-MS and LC-MS/MS data were acquired. LC-MS data were collected from individual samples (*n* = 1 injection), system blanks (injection of solvent used to resolubilize samples), and a pooled quality control (QC). The pooled QC was injected multiple times at different volumes and used in data processing. LC-MS/MS data, used to annotate features, were collected using the AcquireX (Thermo Scientific) deep scan methodology in which the pooled QC was injected multiple times (*n* = 7).

Chromatographic separation was carried out on a 2.1 × 100 mm, 100 Å, 2.6 µm, F5 analytical column (Phenomenex) with corresponding guard cartridge. The column was maintained at 30°C during separation with solvent pre-heater. Gradient elution was performed after an initial period of isocratic elution using water with 0.1% acetic acid vol/vol (A) and acetonitrile with 0.1% acetic acid vol/vol (B). Separation was performed as follows: 0% B from 0 to 2.0 min, 0% to 100% B from 2.0 to 10.5 min, 100% B from 10.5 to 12.0 min, 100% to 0% B from 12.0 to 13.0 min, 0% B from 13.0 to 20.0 min. The flow rate was 0.5 mL/min.

MS and MS/MS data were collected with an EASY-IC. MS data were acquired at 120,000 resolution from *m/z* 100–1,000 with an radio frequency (RF) lens of 60% and maximum injection time of 50 ms. MS/MS data were acquired at 30,000 resolution using an isolation width of 1.5 (*m/z*), stepped-assisted higher energy collisional dissociation (HCD) (energy steps were 20, 35, and 60, and a maximum injection time of 54 ms). The inclusion list was generated and updated via AcquireX with a low and high mass tolerance of 5 ppm. Dynamic exclusions were performed with the following parameters: exclude after *n* = 3 times, if occurs within 15 s, exclusion duration of 6 s, a low mass tolerance of 5 ppm, a high mass tolerance of 5 ppm, and excluding isotopes.

### Untargeted metabolomics data processing

Compound Discoverer 3.3.0.550 (Thermo Scientific) was used to process.raw files to generate descriptors of each feature (e.g., *m/z*, retention time), annotation information (e.g., MS/MS database match), and peak area. We processed the output from Compound Discoverer using in-house R scripts via Jupyter Notebooks in order to format the data, putatively annotate chemical features, assess signal response in pooled QC samples, assess signal variance in the pooled QC versus individual samples (i.e., dispersion ratio), and perform statistical analyses (multivariate and univariate). Features were putatively annotated based on MS/MS spectral matching to in-house reference libraries, publicly available databases, and authentic chemical standards where possible. Representative metabolites have been affirmed by MS/MS matching, *m/z* mass error, and retention time similarity to authentic chemical standard. The affirmation of itaconic acid performed for this study in the positive ion mode to level 1 confidence is shown in Fig. S3.

### Whole ear transcriptomics

Ears were collected on the indicated day post-infection and immediately placed in buffer RLT (Qiagen). Tissues were homogenized, and RNA was isolated using Lysing Matrix S (1/8″) metal beads (MPBiomedicals) and a FastPrep-24 Instrument (MPBiomedicals). RNA was then purified using a Qiagen RNAEasy Mini Kit (Qiagen) according to the manufacturer’s protocol. An on-column DNAse digestion was performed prior to RNA elution. RNA concentration and purity were accessed using an Agilent TapeStation 4200. Samples were sequenced on a NovoSeq 6000 S1 using Illumina Stranded Total RNA Prep, Ligation with Ribo-Zero Plus, and paired-end sequencing. The samples had 142 to 216 million pass filter reads with more than 90% of bases above the quality score of Q30.

Raw fastq files were trimmed for quality and adapter contamination with Cutadapt v1.18 (25). Trimmed reads were aligned to the mouse GRCm38.p6 reference genome and GENCODE release 21 reference transcriptome using STAR v2.7.0f run in two-pass mode (26). RNA-Seq by Expectation-Maximization (RSEM) v.1.3.1 (27) was used to compute the expected counts which were further converted to counts per million (CPM) by eVITTA (28). Genes that were expressed with at least 1 CPM in a minimum of three samples were included in the principal component analysis (PCA) and differential expression testing by edgeR (v.3.40.2) (29). Differential expression analysis compared samples in different conditions including mice receiving OT-I T cells with NPS infection vs no OT-I transfer with NPS, OT-I with NPE vs transferred OT-I with NPS infection, OT-I with NPE vs naïve tissue (control), and transferred OT-I with NPS infection vs naïve tissue (control). False discovery rate (FDR) cut-off value of 0.05 was used to detect differentially expressed genes for downstream analysis. PCA projections and volcano plots were generated using ggpubr (v.0.6.0) and ggplot2 (v.3.4.2) packages in R (v.4.2.2), respectively.

Pre-ranked gene set enrichment analysis (GSEA) was performed by ranking genes based on the *P*-value and log_2_ fold change statistics from the edgeR analysis. Gene Ontology (GO) biological process (BP) GSEA was performed using (30) and Kytoto Encyclopedia of Genes and Genomes (KEGG), and Reactome (RA) pathways GSEA were performed using eVITTA (28). Significant enrichments were those with an FDR *q*-value <0.05. Pathway enrichment map visualizations for glutamate metabolism were performed using Cytoscape (v. 3.9.1) (31).

### Statistical analyses

Statistical significance for individual metabolites was calculated using a univariate *t*-test with false discovery rate correction or one-way analysis of variance (ANOVA) with Dunnett’s multiple comparisons. Statistical analyses and graphs were performed and generated using GraphPad Prism 9, R, or as otherwise indicated in the supplemental Materials and Methods section. PCAs and volcano plots were generated in R using prcomp and ggplot packages. Data used to generate PCAs are provided in Table S1. For volcano plots, *p*-values and log_2_ fold change between groups calculated based on mass spec peak area of all features were used for illustrative graphs.

## RESULTS

### T cells and monocytes are recruited to VACV-infected skin

**Fig 1:**
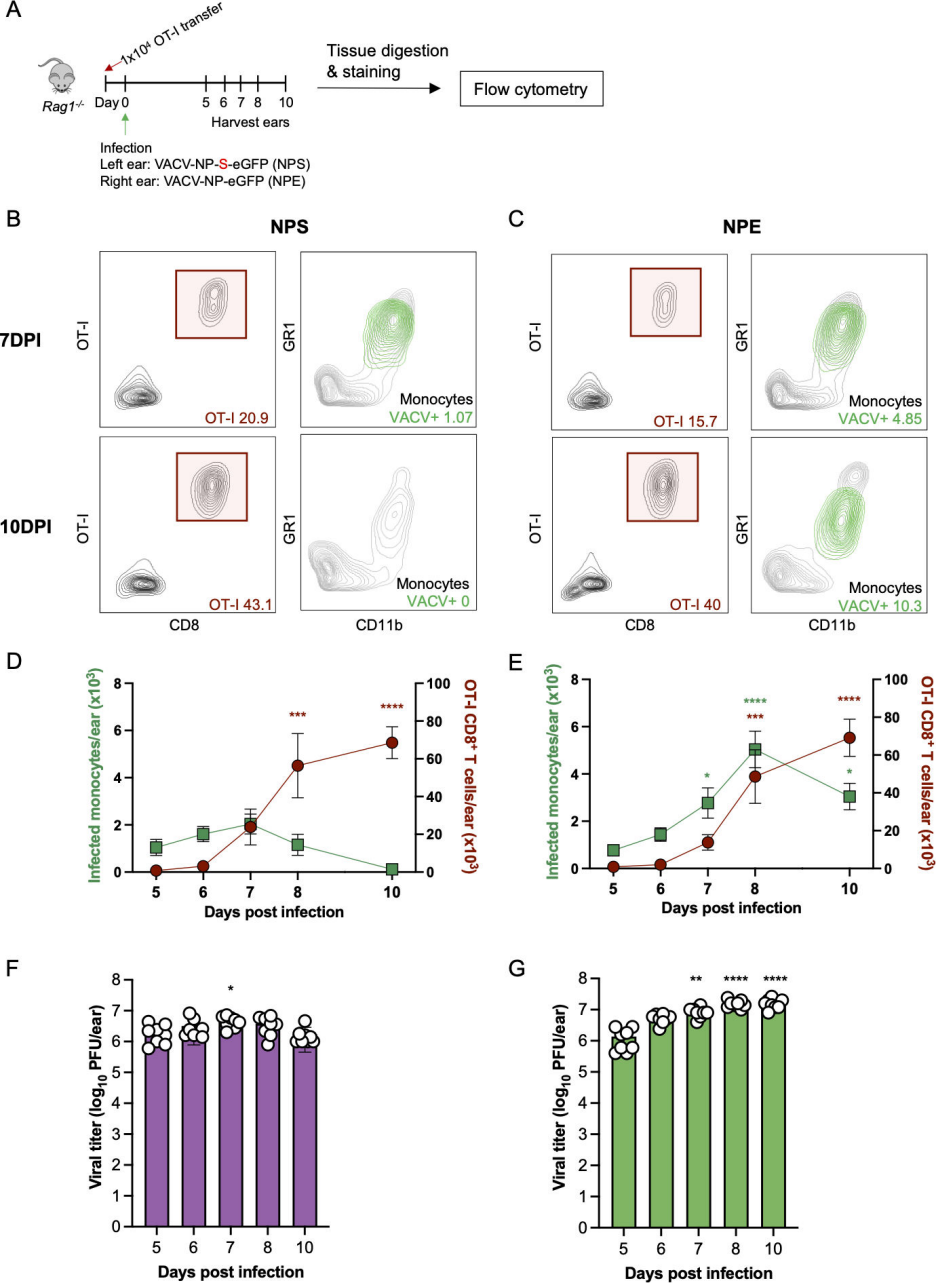
OT-I CD8^+^ T cell killing kinetics of VACV-infected monocytes in the skin.(**A**) Experimental design to study the killing kinetics of adoptively transferred OT-I CD8^+^ T cells in VACV-infected mouse ears. (**B and C**) Representative flow plots from 7 dpi (top panels) and 10 dpi (bottom panels) showing OT-I CD8^+^ T cells (left panels) and VACV-infected monocytes (right panels) overlaid on total live CD45^+^ lymphocytes in (**B**) NPS- and (**C**) NPE-infected ears. Percentages of indicated cell populations are included in the representative plots. (**D and E**) Numbers of OT-I CD8^+^ T cells (red) and infected monocytes (green) following (**D**) NPS and (**E**) NPE infection in ears over five time points post-infection. Dots represent the mean value of all samples (*n* = 8). Error bars show the SEM. Results are from two independent time-course experiments with four animals per time point in each experiment. Statistical analysis was performed using one-way ANOVA and Dunnett’s multiple comparison between each time point post-infection to 5 dpi as the reference group (*P < 0.05, **P < 0.01, ***P < 0.001, ****P < 0.0001). (**F and G**) Viral titers determined by plaque assay for all samples over the five time points post-infection for (**F**) NPS- and (**G**) NPE-infected tissues. Error bars show the SEM. Results are from two independent experiment with four animals per time point in each experiment. Statistical analysis was performed using one-way ANOVA and Dunnett’s multiple comparison between each time point using 5 dpi as the reference group (*P < 0.05, **P < 0.01, ***P < 0.001, ****P < 0.0001).

We have previously established and characterized an immunocompetent mouse model of epicutaneous poxvirus infection with VACV (10, 11, 32). Here, we adapted this model to analyze metabolic changes occurring in VACV-infected skin during T cell-mediated viral clearance. To control T cell killing conditions, we adoptively transferred 1 × 10^4^ OT-I CD8^+^ T cells (TCR transgenic T cells recognizing SIINFEKL) into *Rag1^-/-^* recipients (lacking endogenous adaptive immune cells including T and B cells). In this system, only the adoptively transferred OT-I CD8^+^ T cells can kill SIINFEKL-expressing VACV-infected monocytes. Therefore, to compare tissue in which CD8^+^ T cells could kill infected cells to those without T cell killing, we infected one ear of the mouse with VACV-NP-S-eGFP (expressing SIINFEKL, abbreviated NPS throughout the text) and the other with VACV-NP-eGFP (lacking SIINFEKL, abbreviated NPE). In this model, NPS infection activates OT-I CD8^+^ T cells, which then traffic into both ears regardless of SIINFEKL expression; however, no OT-I CD8^+^ T cell-mediated killing occurs in NPE-infected ears (11). We harvested whole ears at 5, 6, 7, 8, and 10 dpi (Fig. 1A). Flow cytometric analysis of single-cell suspensions of VACV-infected skin revealed detectable numbers of infected monocytes and adoptively transferred OT-I CD8^+^ T cells by 5 dpi and throughout the five study time points (Fig. 1B through E). By 7 dpi, Ag-specific CD8^+^ T cells continued to accumulate in the skin and accounted for ~10%–20% of the total lymphocytes (Fig. 1B and C, top panels). As T cells entered and killed infected cells, NPS-infected monocytes (determined by virus-encoded GFP expression) decreased in number until they were negligible by 10 dpi (Fig. 1B, bottom panel, and Fig. 1D). In the contralateral ear infected with NPE (lacking cognate Ag), VACV-infected monocytes remained high in number throughout day 10 even though both ears had similar number of cytotoxic CD8^+^ T cells in the tissue (Fig. 1C, bottom panel, and Fig. 1E). Despite clearance of infected monocytes by 10 dpi in NPS-infected tissues by OT-I CD8^+^ T cells, infectious virus from whole-tissue digestion was still detectable at this time point (Fig. 1F). However, VACV viral titers gradually increased over this time period in NPE-infected tissue (without cytotoxic T cell restriction) (Fig. 1G). Together these data show that VACV infection of the skin induces T cell recruitment that clears virus-infected monocytes and reduces overall VACV viral titers in an Ag-dependent manner by 10 dpi.

### VACV infection induces metabolic changes in the skin

**Fig 2:**
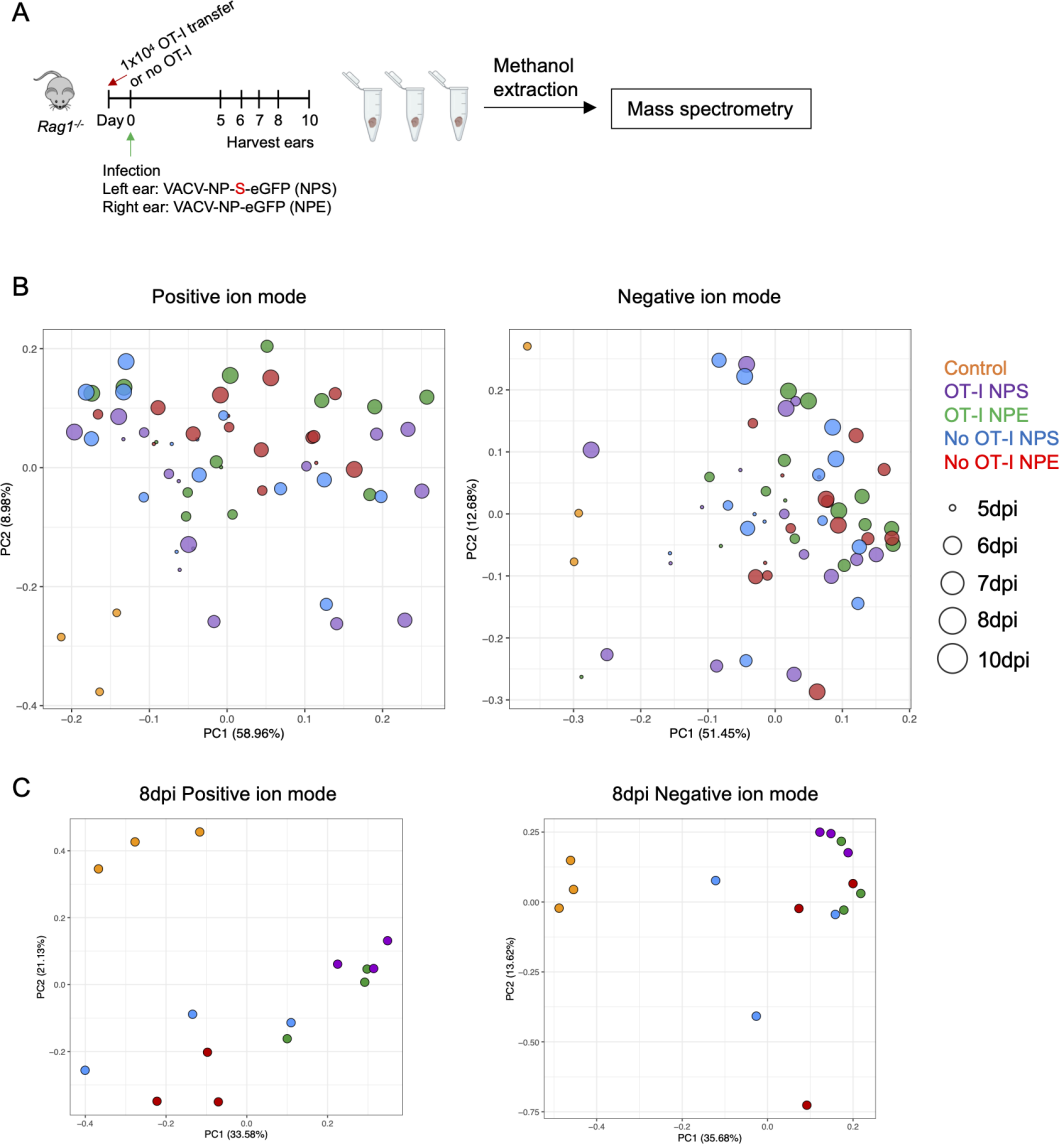
Metabolomics of VACV-infected skin reveals infection-induced changes.(**A**) Experimental design for whole-tissue metabolomics. Ears from five experimental groups were harvested on 5, 6, 7, 8, and 10 dpi and kept frozen until methanol extraction. Methanol-extracted samples were analyzed by untargeted metabolomics. (**B**) PCA plots of features in positive and negative ion mode for all samples. Each data point represents an individual sample (*n* = 3 per condition per time point). Color indicates experimental groups. Circle size reflects the time point of harvest. (**C**) PCA plots of features in positive and negative ion modes for 8 dpi samples. Dots show individual samples. Colors represent experimental groups.

Using this system of infection with two different VACVs, we next sought to examine the metabolic profile of VACV-infected skin in the presence and absence of cytotoxic CD8^+^ T cell-mediated clearance of viral infection. We performed whole-tissue untargeted metabolomics on VACV-infected skin from our infection model (NPS or NPE infection of *Rag1^-/-^* mice) with and without adoptively transferred OT-I CD8^+^ T cells (Fig. 2A). Thus, we included five different sample groups: uninfected naïve mice as controls, NPS infection with transferred OT-I CD8^+^ T cells, NPE infection with OT-Is, NPS infection without OT-Is, and NPE infection without OT-Is. This allowed us to examine metabolic changes induced by T cell entry alone and those associated with viral clearance. Metabolites extracted from whole ear pinna homogenates underwent UHPLC-HRMS/MS in positive and negative ionization mode (measuring polar to moderate non-polar metabolites, such as amino acids and fatty acids, respectively). There were ~350 unique metabolites in the positive ionization mode annotated by MS/MS database and ~170 metabolites annotated in the negative ionization mode. All features (annotated and unannotated) are listed in Tables S1 and S2.

To understand global metabolic changes in the infected skin, we first used PCA to compare the five sample groups based on all features (Fig. 2B). PCA plots composed from features (annotated and unannotated) in both positive and negative ion modes revealed a clear separation of the uninfected control group (orange dots) from the infected samples. Unexpectedly, all post-infection tissue samples displayed very limited separation despite experimental conditions (e.g., the presence or absence of T cells and cognate Ag). Infection progression influenced the tissue metabolome as samples grouped together based on time post-infection. Additionally, the metabolic profile of infection with cognate Ag and Ag-specific T cells was similar to other infected groups and highly different from naïve control tissue. This was also the case when a single time point, 8 dpi, was examined (Fig. 2C), and for all sampled time points (Fig. S2). Thus, VACV infection progression and the ensuing innate immune response were overriding contributors to the overall metabolome of the skin regardless of whether Ag-specific T cells were present in the tissue.

### Metabolic changes align with time post-infection

**Fig 3:**
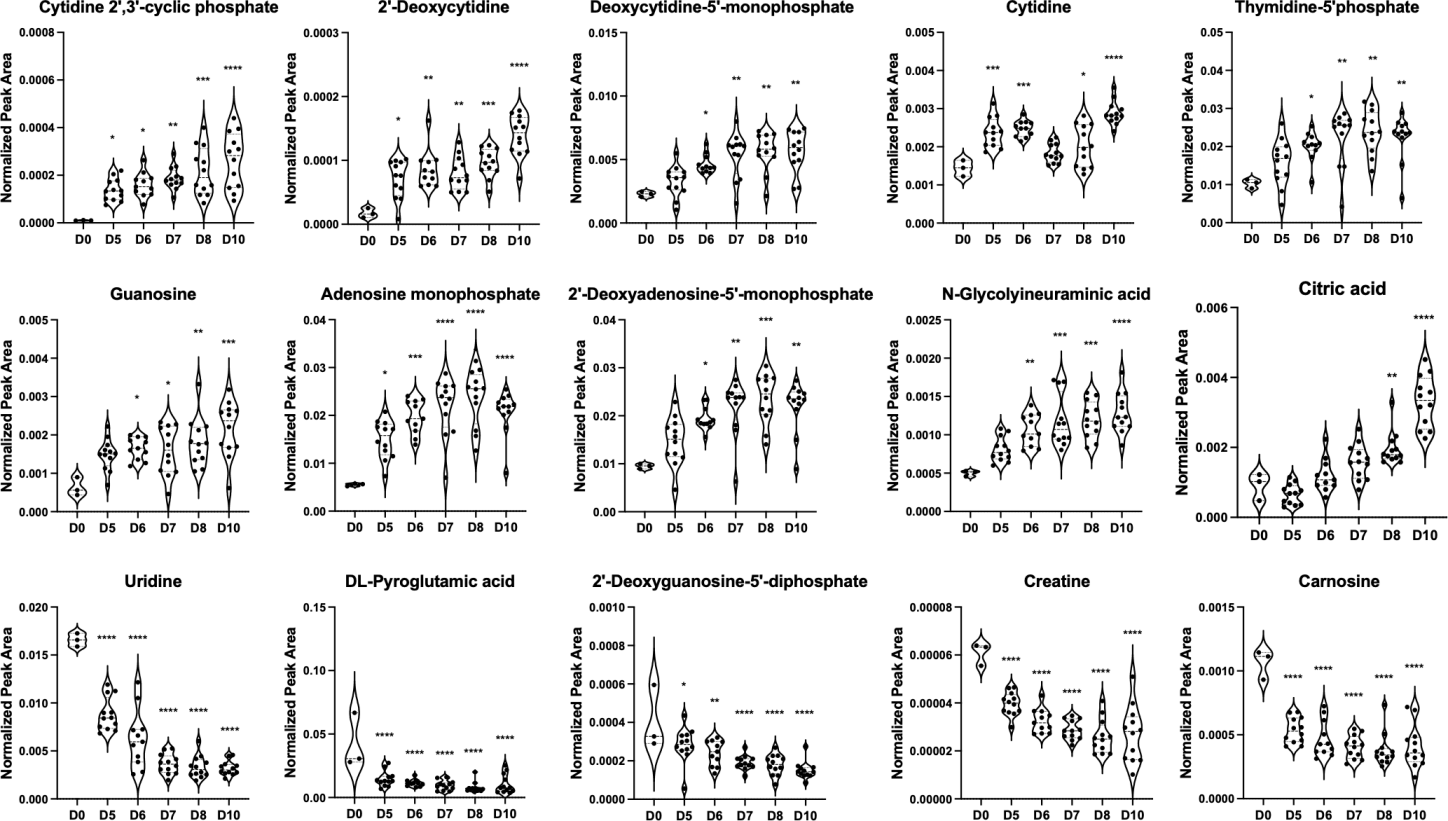
Metabolites in the whole skin reflect infection progression. Selected metabolites with temporal trajectory changes over the infection time course are shown. Representative violin graphs showing 10 metabolites with increased levels over time and five metabolites with reduced levels over time. Dots represent individual samples combined from the five experimental groups. Statistical analysis was performed using one-way ANOVA and Dunnett’s multiple comparison between each time point post-infection to the uninfected group (**D0**) (*P < 0.05, **P < 0.01, ***P < 0.001, ****P < 0.0001).

Although the five experimental groups did not show distinct separation in PCA, a fraction of the total features detected by untargeted metabolomics displayed a gradually increasing or decreasing trajectory (*P* < 0.05 comparing each time point post-infection to the reference time point of 5 dpi) (Fig. 3). We detected increases in several nucleotides and associated molecules involved in DNA synthesis during infection including: deoxycytidine-5´-monophosphate, adenosine monophosphate, 2´deoxycytidine, and guanosine. The changes in nucleotide species over time may reflect the rapid and robust synthesis of the VACV DNA genome that requires large amounts of DNA precursors. We also observed a decreasing trend in several metabolites such as uridine, creatine, and 2´-deoxyguanosine-5´-diphosphate. Interestingly, VACV encodes a uracil DNA glycosylase and a deoxyuridine triphosphatase to prevent uracil incorporation into new DNA genomes (33, 34). Together, our data show that the global metabolome of VACV-infected skin tissue could be largely influenced by time or infection progression.

### Itaconic acid is produced in VACV-infected skin

**Fig 4:**
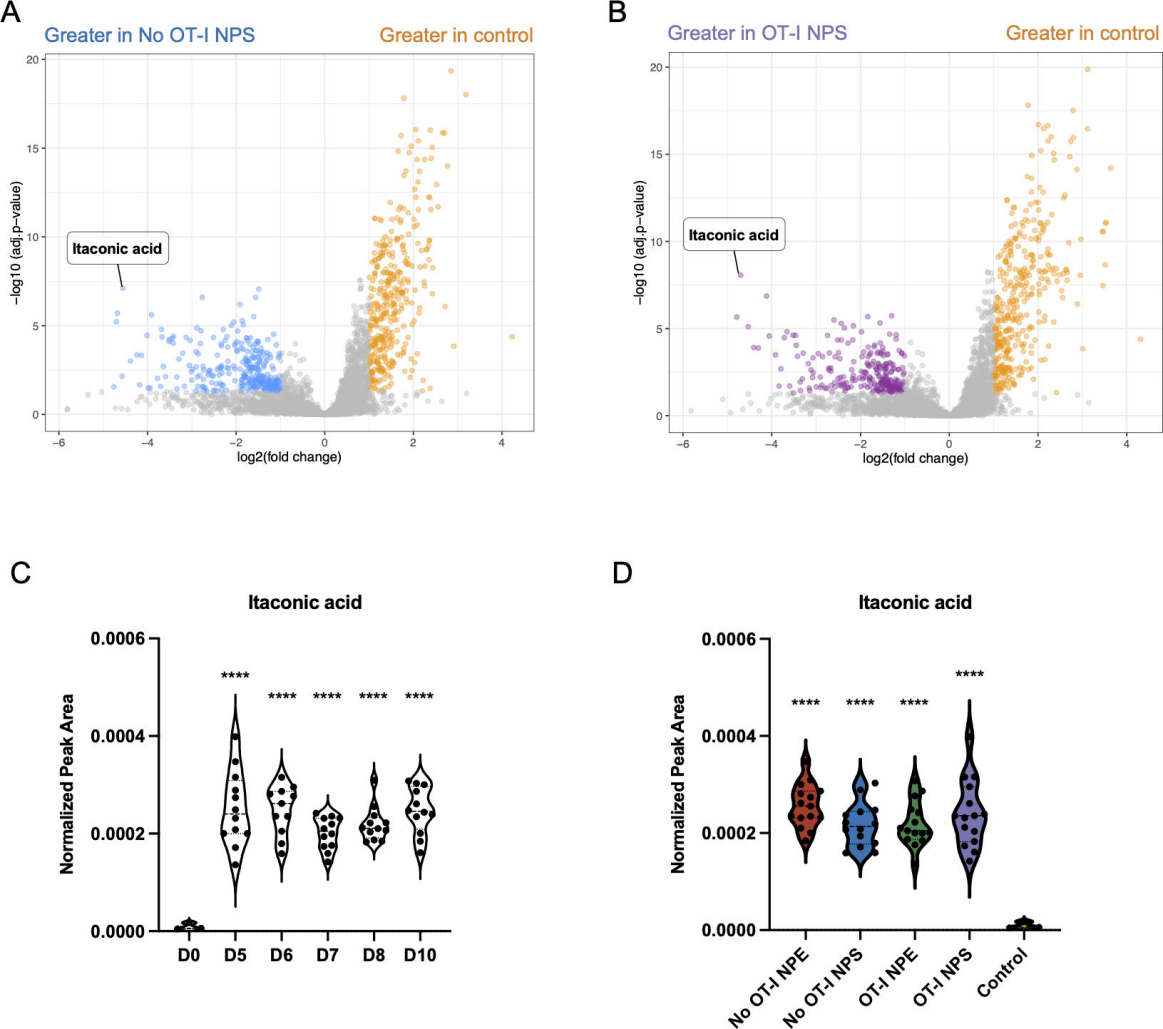
Itaconic acid is produced in VACV-infected skin. (**A and B**) Volcano plot showing significantly different metabolites between (**A**) NPS-infected tissue without transferred OT-I CD8^+^ T cells and uninfected control tissues, and (**B**) NPS-infected tissue with transferred OT-I CD8^+^ T cells and uninfected control samples. Metabolites with adjusted *P*-value of less than 0.05 and a fold change greater than 1 are colored as indicated on the graph. Itaconic acid is labeled. (**C and D**) Violin plots showing itaconic acid levels separated by (**C**) time point (three biological replicates for each sample group on each dpi) and (**D**) experimental groups (*n* = 3 biological replicates per group at each time point). Dots represent individual samples. Statistical analysis was performed using one-way ANOVA and Dunnett’s multiple comparison between each time point or experimental group to the D0 uninfected group (*P < 0.05, **P < 0.01, ***P < 0.001, ****P < 0.0001).

We next sought to assess the magnitude of metabolic changes in the tissue after VACV infection. We compared all features (both annotated and unannotated) from uninfected control tissue to infected samples either without (Fig. 4A) or with (Fig. 4B) OT-I CD8^+^ T cells. In both samples, itaconic acid was identified as a highly upregulated metabolite with the most significant *P*-value compared to uninfected skin. Itaconic acid is produced by myeloid cells and is upregulated during both bacterial and viral infections (35, 36). Itaconic acid was identified via retention time matching, measured accurate mass (*m/z*), and MS/MS spectral information. Potential isobaric metabolites, citraconic acid, and mesaconic acid were excluded based on retention time (Fig. S3). Recently, itaconic acid has been the focus of intense study due to its immunomodulatory functions (21). Therefore, we closely examined itaconic acid levels over time in our samples with different conditions. By 5 dpi, our earliest time point, itaconic acid was significantly upregulated compared to uninfected tissues (*P* < 0.0001) (Fig. 4C). Over the next 5 days of infection, itaconic acid remained highly upregulated ((*P* < 0.0001 for each time point post-infection). Analysis of samples undergoing T cell killing (or not) also did not reveal any clear differences in itaconic acid levels (Fig. 4D). Thus, although T cells kill infected monocytes, elimination of this infected monocyte subpopulation does not appear to be enough to shift the whole-tissue metabolome away from itaconate production. Notably, however, there are still numerous uninfected myeloid cells and other leukocytes present in the ear tissue over the five study time points (Fig. S*4*).

### RNA sequencing of whole skin reveals altered metabolic pathways during infection

**Fig 5:**
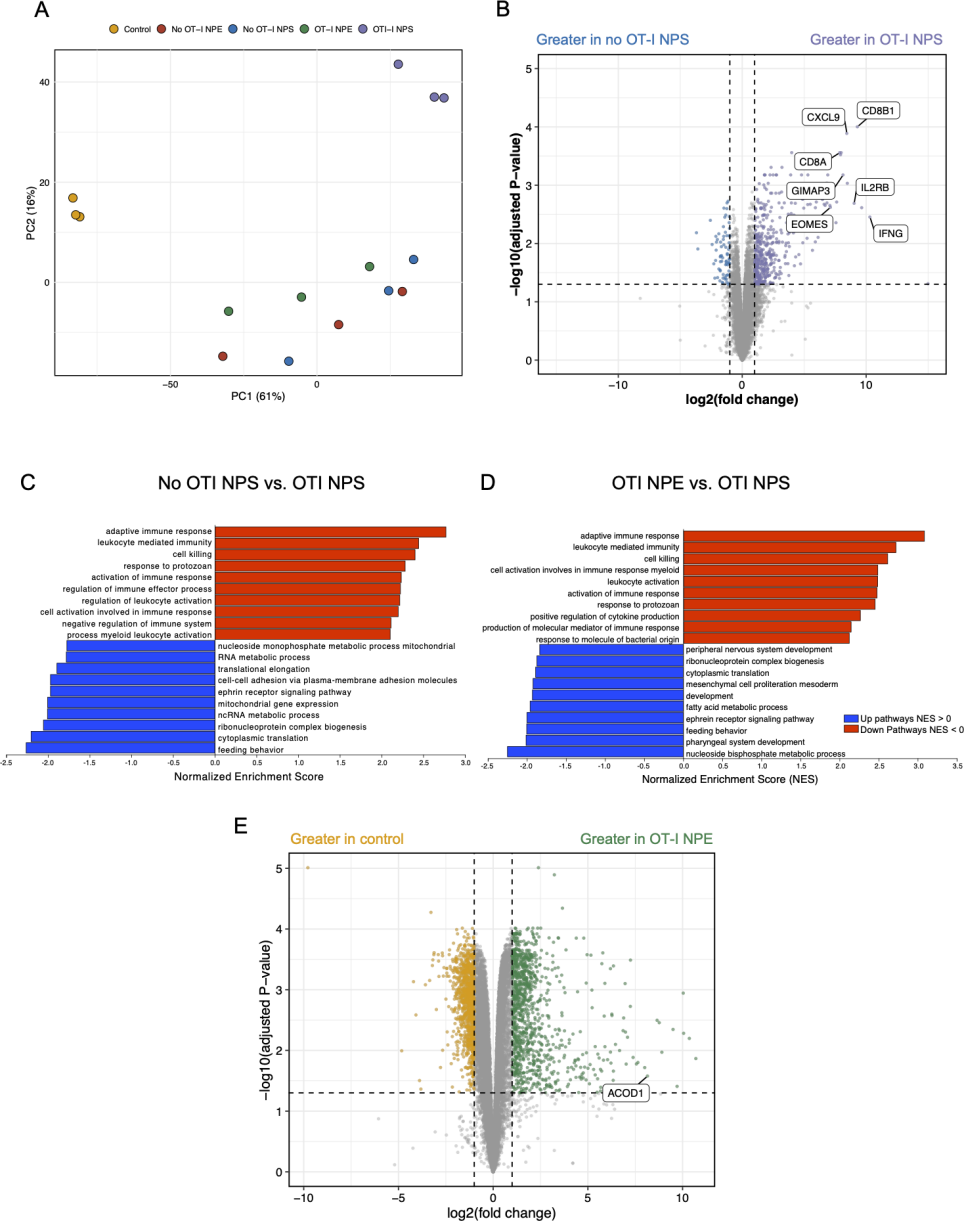
RNA-Seq analysis of VACV-infected skin reveals altered metabolic pathways.(**A**) PCA of NPS- and NPE-infected tissue with and without OT-I CD8^+^ T cells and uninfected controls (three replicates per group) based on gene expression at 6 dpi. Samples are color-coded based on experimental groups. Dots represent individual samples. (**B**) Volcano plot displaying differentially expressed genes (DEGs) in NPS-infected ears without OT-I CD8^+^ T cells compared to those with OT-I CD8^+^ T cells on 6 dpi by log_2_ fold change (x-axis) and adjusted *P*-value (y-axis). Colored and labeled DEGs above the dotted line represent those with FDR *P*-value <0.05 and log_2_ |FC| ≥ |2| which were considered significant. (**C and D**) GSEA was performed using the GO BP term gene set for (**C**) NPS-infected skin with and without OT-I CD8^+^ T cells, and (**D**) NPS-infected versus NPE-infected skin (both with OT-I CD8^+^ T cells). Only significantly (FDR < 0.05) upregulated (blue) and downregulated (red) pathways are shown, represented by normalized enrichment score. (**E**) Volcano plot displaying DEGs in uninfected control tissue compared with NPE-infected skin with transferred OT-I CD8^+^ T cells.

To complement our metabolomics analyses, we performed RNA sequencing of uninfected whole ear tissue as well as infected ear tissue with and without transferred OT-I CD8^+^ T cells at 6 dpi to identify transcriptomic changes associated with VACV infection. In comparison to the metabolome PCAs (Fig. 2), a PCA based on gene expression revealed more distinct separation of the five sample groups. Uninfected control tissues and tissues undergoing T cell-mediated killing clustered separately from groups lacking killing (Fig. 5A). We identified 285 genes that were significantly upregulated (log_2_ |FC| ≥ 1.5, FDR ≤ 0.05) and 31 genes that were significantly downregulated (log_2_ |FC| ≤ −1.5, FDR ≤ 0.05) in NPS-infected tissues with OT-I CD8^+^ T cells compared to NPS-infected tissues without OT-I CD8^+^ T cells. Several T cell-specific genes were significantly upregulated including *Cd8a*, *Cd8b1*, *Il2rb*, *Eomes*, and *Ifng* (Fig. 5B). Additionally, *Gimap3*, which codes for a GTPase located on the outer membrane of the mitochondria, was significantly upregulated in the tissue with cytotoxic T cells (Fig. 5B) (37).

The nature of these changes in expression was interrogated using a pre-ranked GSEA to determine enrichment in GO BP terms and KEGG and RA pathways. GO terms related to adaptive immune response and T cell-mediated cell killing were upregulated in NPS-infected tissues with OT-I CD8^+^ T cells compared to those without OT-I CD8^+^ T cells (Fig. 5C). However, GO terms related to mitochondrial RNA processing and mitochondrial gene expression were upregulated in NPS-infected ears without OT-I CD8^+^ T cells or cytotoxic T cell-mediated control of VACV infection (Fig. 5C). Pathways related to arachidonic acid metabolism (adj. *P*-value = 0.018), pyruvate metabolism (adj. *P*-value = 0.04), mitochondrial translation, elongation, termination (adj. *P*-value = 0.04), and tricarboxylic acid cycle (TCA) and respiratory electron transport (adj. *P*-value = 0.045) were significantly upregulated in NPS tissues without OT-I CD8^+^ T cells (thus due to transcriptional changes in other cells during infection) (Table S4).

We identified many of the same GO BP terms enriched in ears containing OT-I CD8^+^ T cells that were infected with NPS versus NPE (Fig. 5D). The GO BP term “fatty acid metabolic process” was significantly upregulated in NPE-infected tissue (lacking killing) compared to NPS-infected tissues with OT-I CD8^+^ T (Fig. 5D). Several metabolic pathways were significantly upregulated in NPE-infected ears including valine, leucine, and isoleucine degradation (adj. *P*-value = 0.009); propanoate metabolism (adj. *P*-value = 0.009); α-linolenic acid metabolism (adj. *P*-value = 0.015); fatty acid elongation (adj. *P*-value = 0.015); TCA (adj. *P*-value = 0.015); and tyrosine metabolism (adj. *P*-value = 0.03) (Table S4). Although no unique pathways were identified that contained the enzyme responsible for generating itaconic acid (aconitate decarboxylase 1, ACOD1), *Acod1* expression was significantly upregulated (~9-fold, adj. *P*-value = 0.04) in infected skin compared to uninfected controls (Fig. 5E).

Together, these data reveal multiple transcriptional pathways poised to alter tissue metabolism during VACV infection, which corresponded to many of the infection-induced alterations in the tissue metabolome.

### Antiviral T cells alter specific metabolite levels in the skin

**Fig 6:**
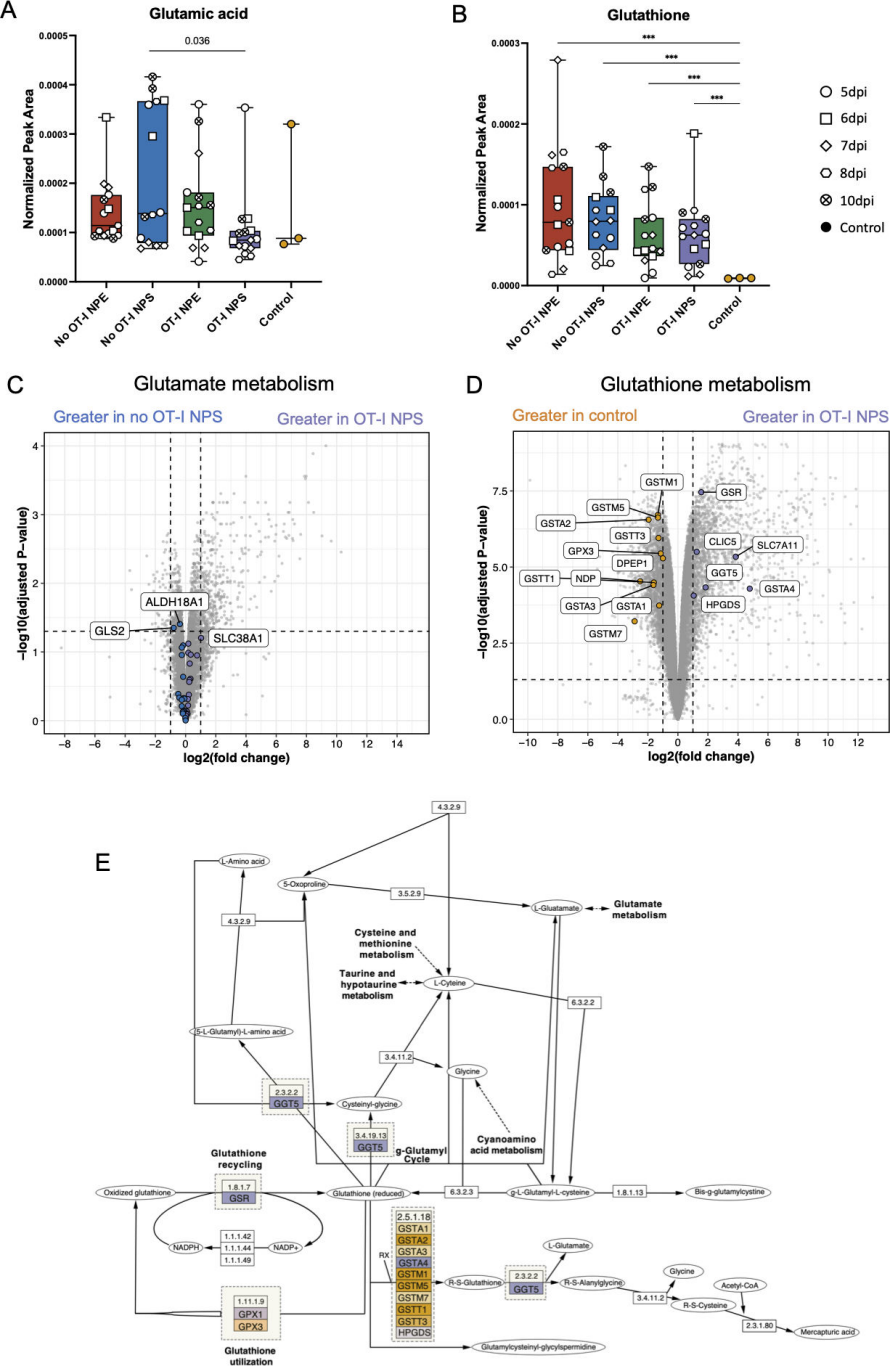
Select metabolites change in the skin during T cell-mediated viral clearance.(**A**) Box plot showing detected levels of glutamic acid. Each data point represents an individual sample. The shapes of the data points indicate the collection time point. Error bars show the minimal to maximal range of the data. Statistical significance was calculated by a multiple *t*-test with Benjamin-Hochberg false discovery rate correction. (**B**) Box plot illustrating glutathione levels detected in each experimental group. The shapes of the data points indicate collection time point. Statistical significance was calculated using one-way ANOVA and Dunnett’s multiple comparison between each experimental group to the uninfected group (*P < 0.05, **P < 0.01, ***P < 0.001, ****P < 0.0001). (**C**) Volcano plot showing differentially expressed genes from RNA-Seq data in the glutamate metabolism pathway in NPS-infected tissue with and without OT-I CD8^+^ T cells. Significantly altered genes in the glutamate metabolism pathway identified in the KEGG database are colored with gene names labeled in boxes. (**D**) Volcano plot of differentially expressed genes in the glutathione metabolism pathway in NPS-infected skin with OT-I CD8^+^ T cells compared to uninfected control tissue. Gene expression changes reaching statistical significance in the glutathione metabolism pathways are color coded and labeled. (**E**) Metabolic pathway map illustrating glutamine/glutathione metabolism and associated protein mediators using Cytoscape. Significantly expressed genes encoding for proteins in the glutamine and glutathione metabolism pathways corresponding to genes identified in (D) are colored. Uninfected control group is shown in yellow and OT-I NPS-infected skin in purple, corresponding to the colors in the volcano plots.

Despite the infection-driven global metabolic changes observed through metabolomics and transcriptomics analyses in whole skin tissue, we detected significant differences in glutamic acid that varied with the adoptive transfer of T cells (Fig. 6A). Glutamic acid was significantly reduced in NPS-infected tissue with OT-I CD8^+^ T cells compared to those without T cells (*P* = 0.036), indicating the possible consumption or utilization of glutamic acid by cytotoxic OT-I CD8^+^ T cells during Ag-specific killing (Fig. 6A). As a product of glutamine metabolism, glutathione was significantly increased in all sample groups compared to uninfected control samples (Fig. 6B), indicating both infection and immune responses could contribute to the production of glutathione.

Given the identification of glutamic acid and glutathione metabolic changes, we explored differentially expressed genes involved in the glutamine and glutathione metabolism pathways comparing mRNA levels from NPS-infected tissues with OT-I CD8^+^ T cells to those without T cells and to uninfected control tissues (Fig. 6C through E). A volcano plot revealed the upregulation of *Gls2*, encoding glutaminase that catalyzes the breakdown of glutamine to glutamate and ammonia, in no OT-I-NPS tissues, consistent with the higher level of glutamic acid detected by metabolomics (Fig. 6A and C). *Slc38A1*, encoding a key glutamine transporter, was also significantly upregulated in NPS-infected tissue with OT-I CD8^+^ T cells (Fig. 6C). Likewise, numerous glutathione metabolism-associated genes were differentially expressed in NPS-infected tissue with T cells compared to uninfected control tissues (Fig. 6D). In particular, the expression of *Gsr,* encoding glutathione-disulfide reductase that is responsible for glutathione recycling, was significantly increased in NPS-infected tissue with T cells (Fig. 6D). Furthermore, mRNA encoding several glutathione S-transferases (*GSTA* genes) were significantly lower in NPS-infected tissue with T cells compared to control samples (Fig. 6D and E).

Collectively, our data show that glutamine and glutathione metabolism, identified by both metabolomics and transcriptomics, are differentially regulated during T cell-mediated VACV clearance in the skin.

## DISCUSSION

With the tremendous recent advances in untargeted metabolomic profiling technologies, it is now possible to analyze complex tissues, such as the skin, to better understand metabolic alterations during infection or disease. Previous studies have characterized the skin metabolome of dermatological disorders and diseases such as psoriasis, atopic dermatitis, and melanoma (38, 39). The identification of specific amino acid and lipid metabolites in these inflammatory skin disorders have demonstrated the potential value of using metabolic biomarkers as diagnostic and prognostic tools. However, studies of the whole-tissue metabolome during cutaneous viral infections have been lacking. Here, we used whole-tissue mass spectrometric metabolomic profiling and RNA sequencing to identify metabolic pathways altered in the skin during cutaneous VACV infection. We observed changes that were likely due to the metabolic requirements of the virus as well as due to myeloid and T cells recruited into the skin to help control viral replication.

Untargeted metabolomic profiling revealed several unique features of poxvirus-infected skin. The global metabolomic signature of VACV-infected skin tissue was clearly distinct from uninfected skin, with several metabolites, including itaconic acid, upregulated after infection. While we expected CD8^+^ T cell-mediated killing of VACV-infected cells to shift the metabolomic profile in the tissue, we did not uncover significant differences in the levels of most metabolites in experimental groups with and without OT-I CD8^+^ T cells. Instead, progression of infection and the innate immune response overwhelmingly shaped the skin metabolome during infection. Levels of many metabolites in nucleic acid metabolism such as adenosine monophosphate, 2´-deoxycytidine, guanosine, and uridine were altered in a time-dependent manner. VACV replicates to create roughly 10,000 new DNA genomes of approximately 200 kb each per infected cell, incurring considerable nucleotide consumption (40, 41). Furthermore, VACV encodes several viral DNA-modifying enzymes, including uracil-DNA glycosylase and thymidine kinases, that catalyze DNA repair and the rapid generation of DNA precursor pools (42, 43). Finally, in addition to viral replication, VACV inhibits and interferes with host DNA synthesis, mRNA processing, and protein translation, which may result in the diminishing levels of metabolites such as uridine (9). Therefore, VACV possesses many known mechanisms that could account for the perturbed nucleic acid metabolism we observed in infected skin.

One of the most upregulated metabolites in our metabolomics data was itaconic acid. Itaconic acid is produced by myeloid cells, including monocytes and macrophages, and is the product of the mitochondrial enzyme ACOD1 (21, 36, 44). Cutaneous VACV infection induces a large influx of monocytes into the skin (10), and *Acod1* mRNA was highly upregulated in RNA sequencing data from whole skin. *In vitro*, the TCA cycle intermediate aconitate is increased in VACV-infected fibroblasts (45), suggesting that infected monocytes might also have access to increased precursors for conversion to itaconic acid. Although further metabolic profiling will need to be performed on monocytes isolated from the infected skin, our data suggest that monocytes respond to the inflammatory environment during VACV infection through the production of itaconic acid. Interestingly, this pathway has recently been shown to exert multiple immunomodulatory functions during antiviral immune responses. For instance, itaconic acid can inhibit M2 macrophage polarization *in vivo* (46). Itaconic acid regulates production of the key antiviral cytokines type I interferons (IFN-I) (47). As a result, itaconic acid may also exhibit antiviral activity that is independent of its role in IFN-I production (21, 48). Lastly, itaconic acid is also thought to be anti-inflammatory (21), and skin inflammation, such as that occurring during atopic dermatitis, promotes VACV replication (49, 50). Thus, our untargeted metabolomics approach identified an anti-inflammatory metabolite that could potentially dampen VACV replication or spread in the skin.

Additionally, select metabolites in the glutamine and glutathione metabolism pathways were significantly altered in VACV-infected skin. Glutamic acid was significantly decreased in NPS-infected skin (with Ag-specific OT-I CD8^+^ T cells) compared to those infected with NPE (lacking T cell-mediated killing of infected cells). Glutamic acid, or its ionic form glutamate, is the product of glutamine hydrolysis catalyzed by the enzyme glutaminase. From our RNA sequencing analysis, we observed the upregulation of the glutaminase-encoding gene, *Gls2*, in NPS-infected tissues without OT-I CD8^+^ T cells compared to those with OT-Is, suggesting the possible increase of glutamine hydrolysis in infected tissues lacking T cell-mediated killing. This is consistent with previous *in vitro* studies demonstrating that VACV uses glutamine (and not glucose) for viral replication (5–7, 51), which would be higher in tissues lacking killing (with higher viral titers). Furthermore, glutamate accumulates in VACV-infected cells due to glutamine metabolism and nucleotide biosynthesis, which may reflect our finding of higher glutamic acid levels in NPE-infected tissues that lack Ag-specific T cells.

Several observations suggest that T cells also directly influence glutamine levels in the tissue. The increase of glutamine transporter *SLC38A1* in NPS-infected tissue with OT-I CD8^+^ T cells may indicate the transport and utilization of glutamine during Ag-specific viral elimination. Previous studies have identified glutamine as a key metabolite that fuels T cell activation and function (17, 52). T cells are highly sensitive to glutamine levels *in vitro,* and the precursors or products of glutamine metabolism are unable to replace glutamine (53). In addition to glutamine metabolism, glutathione was increased in all experimental groups compared to uninfected controls. Activation of T cells and viral infections are frequently associated with increased production of reactive oxygen species (54, 55). Glutathione is an endogenous cellular antioxidant that can ameliorate oxidative damage (56, 57). Furthermore, a previous study showed that ablation of T cell glutathione production decreases antiviral immunity against murine lymphocytic choriomeningitis virus infection (56). In the context of metabolic pathways, glutathione synthesis utilizes glutamate as precursors, further highlighting the involvement of glutamine and glutathione metabolism during the T cell response to cutaneous VACV infection.

Several *in vitro* studies have examined the metabolic changes that occur in VACV-infected cells, allowing a direct comparison to our observations *in vivo*. Viral growth factor (VGF) is a VACV-encoded secreted epidermal growth factor (EGF) homolog that enhances viral spread and cellular motility (45, 58–60). As VGF-induced cellular proliferation imposes increased energetic requirements which are fueled through the TCA cycle (5, 61), VGF is a key regulator of host metabolism in infected cells (45). Future studies using a VGF-deletion virus will be needed to understand the effect of VGF *in vivo* on host cell metabolism, but the decreased *in vivo* replication of this virus will also need to be considered in relation to metabolic alterations (58). Unfortunately, we did not identify metabolic components of the TCA cycle in our analyses, so we are unable to quantify the contribution of increased TCA cycle metabolism by VACV-infected, VGF-producing cells.

There were several limitations to our study. First, the discriminatory power of untargeted metabolomics for stereoisomers (and sometimes constitutional isomers) is limited; therefore, annotated metabolites and metabolic pathways should be validated using more specific analytical methods. Second, we did not employ mouse models deficient in the metabolic pathways identified to ascertain the biological role of differentially expressed metabolites during VACV infection. As metabolites were extracted from whole tissue, we were unable to distinguish the levels of extracellular metabolites and intracellular metabolites to determine consumption or synthesis. Lastly, the post-infection time points used in this study were limited to 10 dpi. Later time points after infection clearance would provide better understanding of the metabolic state of tissue recovery.

In this study, we explored the dynamic balance of viral metabolic needs and immune cell requirements in poxvirus-infected skin using whole-tissue metabolomics and transcriptomics. Our data revealed that viral infection and immune response progression were the main factors shaping global metabolic changes in the skin, rather than the ability of T cells to clear virus-infected cells. The complexity of the whole-tissue environment and cellular dynamics will likely mask subtle metabolic differences that might be uncovered with single-cell metabolome analyses. Nonetheless, our whole-tissue analysis provides a framework for better understanding the global metabolic changes that take place in the skin during acute viral infection.

## Data Availability

All data are present in the paper or in supplemental files. RNA-Seq data were deposited in GenBank under accession number GSE236603. Please direct additional data requests to the corresponding author.

## SUPPLEMENTAL MATERIAL

Table S1_negative mode jvi.01272-23-s0001.txt Metabolomics raw data for negative mode.

Table S2_negative mode jvi.01272-23-s0002.txt Metabolite annotations for negative mode.

Supplemental figures and methods jvi.01272-23-s0003.docx Fig. S1 to S4 and supplemental methods on metabolomics.

Table S1_positive mode jvi.01272-23-s0004.txt Metabolomics raw data for positive mode.

Table S2_positive mode jvi.01272-23-s0005.txt Metabolite annotations for positive mode.

Table S3 jvi.01272-23-s0006.xlsx Detailed metabolite information.

Table S4 jvi.01272-23-s0007.xlsx Preranked gene set enrichment analysis of KEGG and Reactome pathways.

ASM does not own the copyrights to Supplemental Material that may be linked to, or accessed through, an article. The authors have granted ASM a non-exclusive, world-wide license to publish the Supplemental Material files. Please contact the corresponding author directly for reuse.
