# Supplementary material for: Profiling whole-tissue metabolic reprogramming during cutaneous poxvirus infection and clearance
Source: J Virol. 2023 Nov 27;97(12):e01272-23. doi: 10.1128/jvi.01272-23 (PMC10734417; doi:10.1128/jvi.01272-23)
Supplement: Supplemental figures and methods — Fig. S1 to S4 and supplemental methods on metabolomics. [file jvi.01272-23-s0003.docx]

**Supplemental Figures**

**
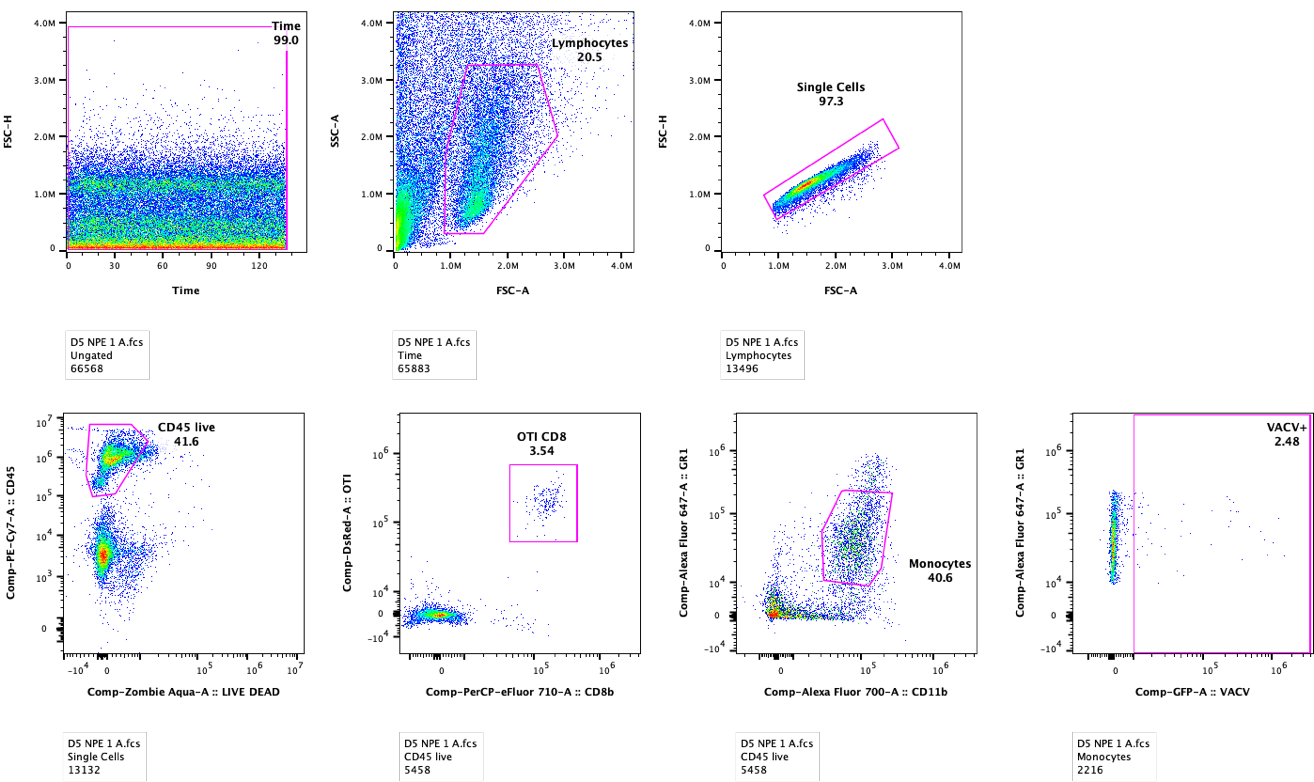
**

**Supplemental Figure 1. Gating Strategy**

Sequential gates to quantitate the percentages of OT-I CD8^+^ T cells, monocytes, and VACV-infected monocytes.


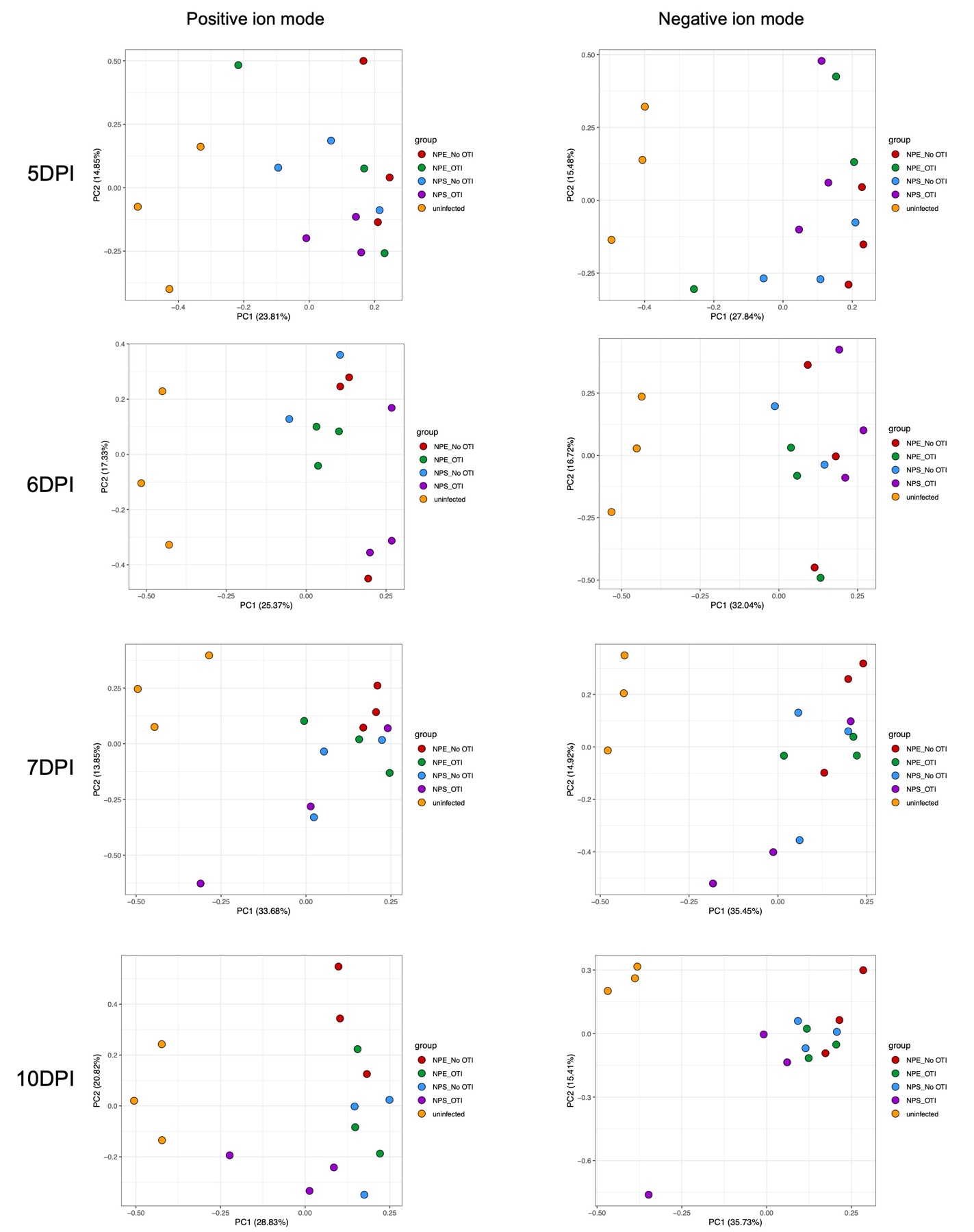


**Supplemental Figure 2. Metabolite PCA by sample time point**

PCA plots of all features identified in the positive and negative ion modes on 5, 6, 7, and 10 dpi. Dots show individual samples. Colors represent experimental groups.


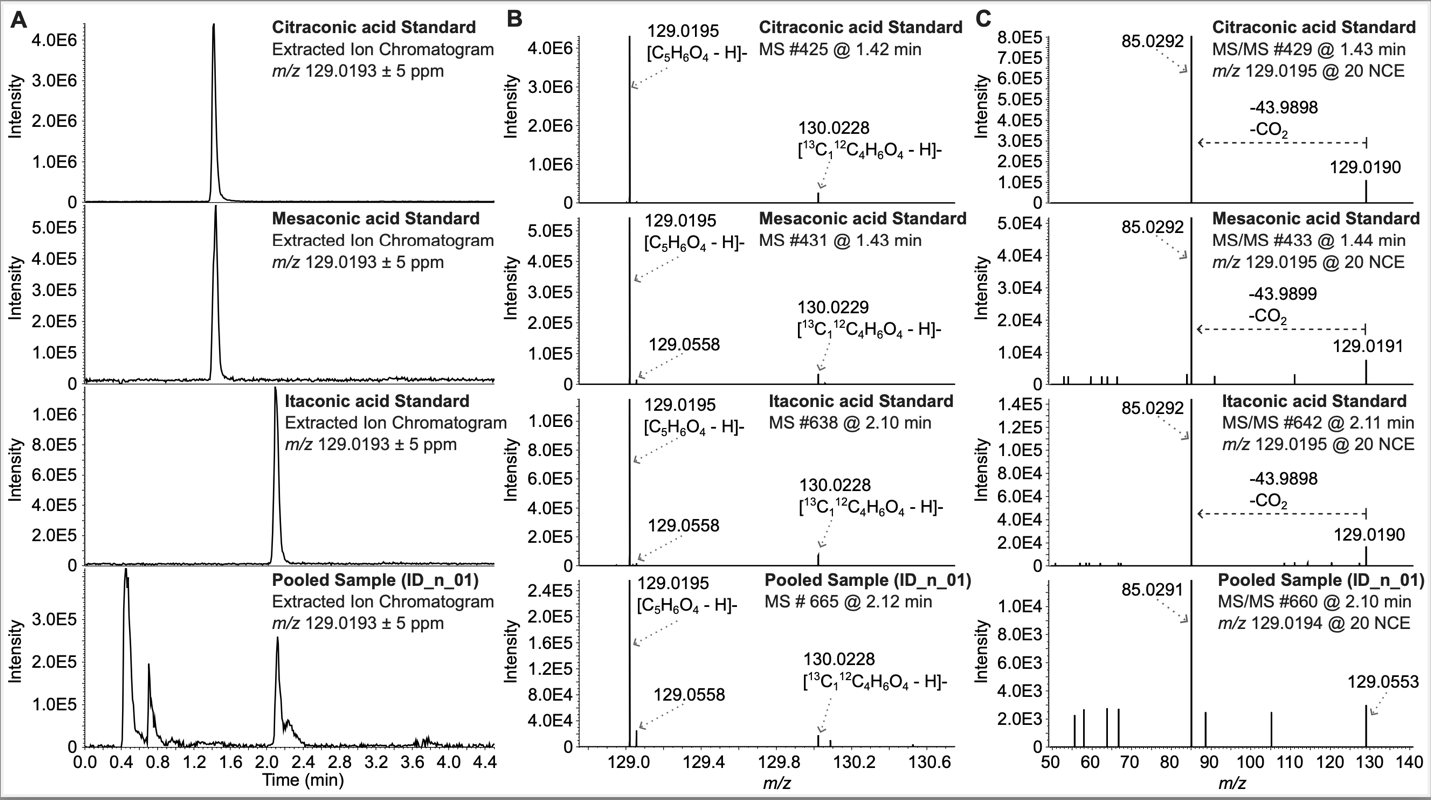


**Supplemental Figure 3. Itaconic acid target validation**

**(A)** Extracted ion chromatogram for *m/z* 129.0193 +/- 5 ppm for citraconic acid, mesaconic acid, itaconic acid, and the pooled sample (ID_n_01). Itaconic acid was identified via retention time match between authentic chemical standard (2.10 min) and pooled sample (2.12 min) **(B)** MS scan (*m/z* 128.75 – 130.75) containing the monoisotopic and 13C peak associated with Citraconic acid, Mesaconic acid, Itaconic acid, and the pooled sample (ID_n_01). *m/z* 129.0195 was observed in citraconic acid, mesaconic acid, and itaconic acid standards matching the theoretical monoisotopic mass for C5H6O4 (measured accurate mass error was 1.55 ppm) for the deprotonated species ([M-H]-). This *m/z* was observed at the same retention time for itaconic acid and the pooled sample, while observed at an earlier retention time for citraconic acid and mesaconic acid. (C) MS/MS spectrum obtained from Citraconic acid, Mesaconic acid, Itaconic acid, and the pooled sample (ID_n_01) isolating the monoisotopic peak and performing higher-energy collision induced dissociation at 20 normalized collision energy (NCE). All data presented were obtained in the negative ionization mode. The MS/MS data obtained from standards and the pooled sample all contained the characteristic product ion *m/z* 85.0292 which results from the loss of CO2. The similarity in structure resulted in similarity in the MS/MS spectral patterns, and was not distinguishing of the small organic acids on its own. However, the measured information, namely the match in retention time, supports the identification of itaconic acid in the negative mode data associated with feature 151|129.01944|2.137.

**
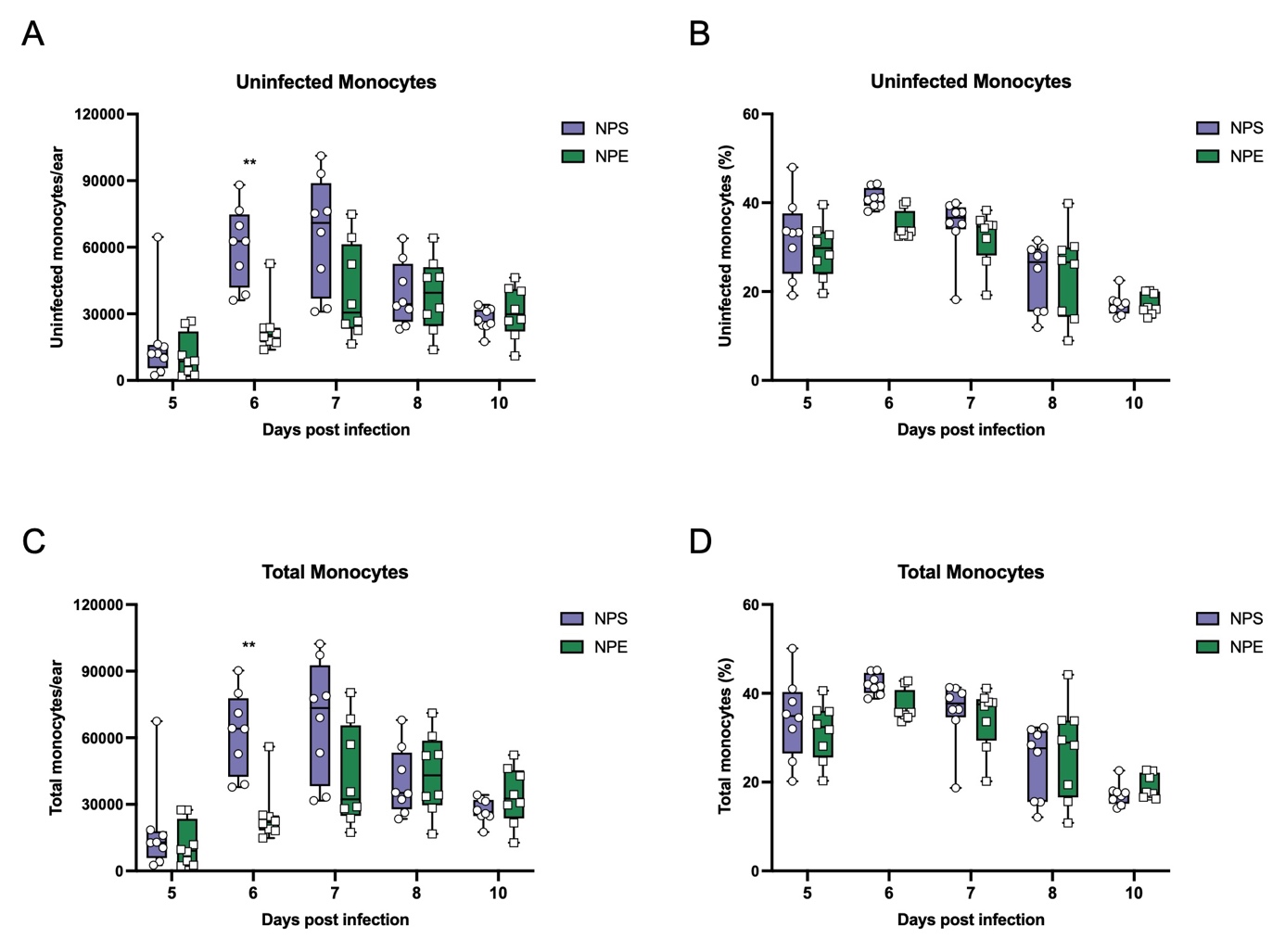
**

**Supplemental Figure 4. Monocyte kinetics during cutaneous VACV infection**

**(A-B)** Summary graphs showing the (A) count and (B) percentage of uninfected monocytes in NPS- and NPE-infected ears over 5 time points post VACV infection. **(C-D)** The (C) count and (D) percentage of total monocytes in NPS and NPE-infected ears over all study time points. Results are from two independent time-course experiments with 4 animals per timepoint in each experiment. Error bars represent the min to max range of all samples. Statistical analysis was performed using one-way ANOVA and Tukey’s multiple comparison between all sample groups and all time points. Statistical significance is only shown for NPS and NPE comparison at each time point (**p*<0.05, ***p*<0.01, ****p*<0.001, *****p*<0.0001).

**Supplemental Materials and Methods**

**1** **Pre-Analytical Materials and Methods**

**1.1** **Sample Preparation**

Extracts were shipped to the Metabolomic Core Facility (NIEHS) overnight on dry ice. Upon arrival the extracts were stored at -80°C until analysis. Dried extracts were removed from storage at -80°C, allowed to warm to room temperature, and resuspended via the addition of 150 uL of water-acetonitrile 98%:2% *v/v.* Resuspended extracts were briefly vortexed (3 s) and then centrifuged for 10 minutes at 14000 rcf at 4°C (Eppendorf Centrifuge 5425R). 50uL of supernatant of the resuspended extract, the soluble fraction of the extract, was transferred to 2 mL autosampler vial (12 mm x 32 mm height vial, 12 mm screw cap, and PTFE/silicone septa, Agilent) containing a microvolume insert (Agilent). The pellet (insoluble extract) was discarded in accordance with chemical and biological safety procedures.

**2** **Analytical Materials and Methods**

**2.1** **Untargeted Metabolomics: Vanquish – Tribrid Fusion**

**Instrumentation**

Samples were analyzed using an ultra-high performance liquid chromatograph (Vanquish^TM^ Horizon UHPLC, Thermo Scientific) coupled to a high-resolution mass spectrometer (Orbitrap Fusion^TM^ Tribrid, Thermo Scientific). EASY-Max NG^TM^ was used as the ionization source, operated in the heated-electrospray ionization (H-ESI) configuration. The source parameters in positive ionization mode were as follows: spray voltage of +4000 V, sheath gas of 50 arbitrary units (arb), auxiliary gas of 10 arb, sweep gas of 1 arb, ion transfer tube at 325°C, vaporizer at 350 °C. The source parameters used in negative ionization mode were identical, with the exception of the spray voltage of -3000 V. Prior to measurement, the mass spectrometer was calibrated using FlexMix (Thermo Scientific) following manufacture directions. EASY-IC™ (Thermo Scientific) was used during data collection; this is a secondary reagent ion source, yielding fluoranthene ions that are used as a lock mass to improve *m/z* accuracy by correcting for mass errors that result from variation in *m/z* measurement (*e.g.* scan-to-scan variation) and environmental changes (*e.g.* ambient temperature)*.*

Chromatographic separation was carried out on a Kinetex F5 analytical column (2.1 inner diameter, 100 mm length, 100 Å, 2.6 µm particle size, Phenomenex) with corresponding guard cartridge. The column was maintained at 30°C during separation. The Vanquish solvent pre-heater was maintained at 30°C. Gradient elution was performed after an initial period of isocratic elution using water with 0.1% acetic acid *v/v* (A) and acetonitrile with 0.1% acetic acid *v/v* (B). Separation was performed as follows: 0% B from 0 - 2.0 min, 0% to 100% B from 2.0 to 10.5 min, 100% B from 10.5 to 12.0 min, 100% to 0% B from 12.0 to 13.0 min, 0% B from 13.0 to 20.0 min. The flow rate was 500 µL min^-1^. A 10 µL static mixer was used. The post column flow path consisted of a Viper^TM^ connection (0.1 mm ID, 550 mm length, Thermo Scientific) from the column to a six-port value and PEEK tubing from the six-port value to the ionization source.

**Data Acquisition: Liquid chromatography – mass spectrometry (LC-MS)**

LC-MS data were collected from individual samples, system blanks, and a pooled quality control. System blanks consist of the same solvent used to resolubilize samples without biological material, and are used to evaluate the chemical background of the results from the analysis system; system blanks were analyzed after every eight samples in the data acquisition order. The pooled quality control (QC) was generated by combining 30uL aliquots from every sample in the data set into one pooled sample. Samples and system blanks were each injected at 4 µL; the QC pool was injected 9 times at different volumes (3 injections each at 2 µL, 4 µL, and 6 µL). MS data were collected with an anticipated LC peak width of 8 s and a default charge of 1. MS data were acquired at 120,000 resolution from *m/z* 100-1000 with an RF lens of 60% and maximum injection time of 50 ms.

**Data Acquisition: Liquid chromatography – tandem mass spectrometry (LC-MS/MS)**

Prior to acquiring LC-MS data for samples, liquid chromatography – tandem mass spectrometry (LC-MS/MS) data were acquired using the AcquireX (Thermo Scientific) deep scan methodology. The acquisition order for AcquireX was the following: system blank (n=1 injection) for exclusion list generation, QC (n=1 injection) for inclusion list generation, and n=7 injections of QC. MS and MS/MS data were collected with an anticipated LC peak width of 8 s and a default charge of 1. MS data were acquired at 120,000 resolution from *m/z* 100-1000 with an RF lens of 60% and maximum injection time of 50 ms. MS/MS data were acquired at 30,000 resolution using an isolation width of 1.5 (*m/z*), stepped assisted higher-energy collision induced dissociation was used with energy steps of 20, 35, and 60 normalized collision energy, and a maximum injection time of 54 ms. The inclusion list was generated and updated via AcquireX with a low and high mass tolerance of 5 part-per-million (ppm) mass error. An intensity filter was applied with an intensity threshold of 2.0 x 10^4^. Dynamic exclusion was used with the following parameters: exclude after n = 3 times; if occurs within 15 s; exclusion duration of 6 s; a low mass tolerance of 5 ppm mass error; a high mass tolerance of 5 ppm mass error; and excluding isotopes.

**3** **Data Processing**

**3.1** **Processing of Raw Data – Feature Finding**

Data files (.raw) were processed with Compound Discoverer 3.3.0.550 (ThermoFisher Scientific) to identify unique molecular features and, where possible, annotate them with chemical names. Features with distinct measured accurate mass, unique retention time, and MS/MS data were tabulated after removal of isotope peaks, blank contaminants, and noise artifacts from the data (workflow and parameters displayed in **Table 1**). The table contained feature descriptors (*e.g. m/z* and retention time), annotation information (e.g. MS/MS database match), and peak area. Features were then processed using R via Jupyter Notebook. Processing steps included formatting of the table output from Compound Discoverer, comparison of *m/z* and retention time of annotated features versus an in-house generated list of *m/z* and retention time based on authentic chemical standards, assessment of signal response in pooled QC samples, assessment of signal variance in pooled QC samples versus samples (i.e. dispersion ratio), and multi- and univariate statistics.

**Table 1.** Compound Discoverer 3.3.0.550 nodes and parameters (in order by row).

| Node | Filter | Parameters |
| --- | --- | --- |
| Select Spectra | Spectrum Properties Filter | Lower RT Limits = 0; Upper RT Limit = 0; First Scan= 0; Last Scan = 0; Ignore Specified Scans = null; Lowest Charge State = 0; Highest Charge State = 0; Minimum Precursor Mass = 100 Da; Maximum Precursor Mass = 1000 Da; Total Intensity Threshold = 0; Minimum Peak Count = 1 |
|  | Scan Event Filters | Mass Analyzer = Any; MS Order = Any; Activation Type = Any; Minumum Collision Energy = 0; Maximum Collision Energy = 1000; Scan Type = Any; Polarity Mode = Any; MS1 Mass Range = null; FAIMS CV = null |
|  | Peak Filters | S/N Threshold (FT=only) = 1.5 |
|  | Replacement for Unrecognized Properties | Unrecognized Charge Replacements = 1; Unrecognized Mass Analyzer Replacements = ITMS; Unrecognized MS Order Replacements = MS2; Unrecognized Activation Type Replacements = CID; Unrecognized Polarity Replacements = +; Unrecognized MS Resolution@ 200 Replacement = 60000; Unrecognized MSn Resolution@200 Replacement = 30000 |
|  | General Settings | Precursor Selection = Use MS(n-1) Precursor; Use Isotope Pattern in Precursor Reevaluation = True; Provide Profile Spectra = Automatic; Store Chromatograms = False |
| Align Retention Times | General Settings | Alignment Model = Adaptive curve; Alignment Fallback = Use Linear Model; Maximum Shift [min] = 0.5; Shift Reference File = True; Mass Tolerance = 5 ppm; Remove Outlier = True |
| Export Spectra | Output Data | Export Format = Mascot Generic Format (*.mgf) |
| Detect Compounds | General Settings | Mass Tolerance [ppm] = 5; Minimum Peak Intensity = 10000; Minimum # Scans per Peak = 5; Use Most Intense Isotope Only = True |
|  | Trace Detection | Maximum Number of Gaps to Correct = 2; Minimum Number of Adjacent Non-Zeros = 2 |
|  | Peak Detection | Chromatographic S/N Threshold = 1.5; Remove Baseline = False; Gap Ratio Threshold = 0.35; Maximum Peak Width [min] = 1.0; Minimum Relative Valley Depth = 0.1 |
|  | Isotope Pattern Detection | Group Isotopes for = Br and Cl; Use Peak Quality for Isotope Grouping = True; Filter out Features with Bad Peaks Only = True; Zig-Zag Index Threshold= 0.2; Jaggedness Threshold = 0.4; Modality Threshold = 0.9; Remove Potentially False Positive Isotopes = True |
|  | Compound Detection | Ions = [M+H]+1; [M+H-H2O]+1; [M+H-NH3]+1; [M+Na]+1; [M+NH4]+1; [M-H]-1; [M-H+HAc]-1; [M-H-H2O]-1; Base Ions = [M+H]+1; [M-H]-1; Remove Singlets = True |
|  | AcquireX Settings | Detect Persistent Background Ions = False |
| Group Compounds | General Settings | Mass Tolerance = 5 ppm; RT Tolerance [min] =0.25; Align Peaks = False; Preferred Ions = [M+H]+1; [M-H]-1; Area Integration = Most Common Ion |
|  | Peak Rating Contributions | Area Contribution = 3; CV Contribution = 10; FWHM to Base Contribution = 5; Jaggedness Contribution = 5; Modality Contribution = 5; Zig-Zag Index Contribution = 5 |
|  | Peak Rating Filter | Peak Rating Threshold = 0; Number of Files = 2 |
| Fill Gaps | General Settings | Mass Tolerance = 5 ppm; S/N Threshold = 1.5; Use Real Peak Detection = True |
| Search mzVault | Search Settings | mzVault Library = in-house database, NIST2020, GNPS (access 03-07-2022), mzCloud Offline for Endogenous 2020B; Compound Classes = All; Match Ion Activation Type = False; Match Ion Activation Energy = Any; Ion Activation Energy Tolerance = 20; Match Ionization Method = False; Apply Intensity Threshold = True; Remove Precursor Ion = True; Precursor Mass Tolerance = 5 ppm; FT fragment Mass Tolerance = 10 ppm; IT Fragment Mass Tolerance = 0.4 Da; Match Analyzer Type = False; Search Algorithm = HighChem HighRes; Match Factor Threshold = 50; Maximum Number Results = 10; RT Tolerance [min] = 0.5; User Retention Time = False |
| Search Mass List | Search Settings | Mass Lists = in-house mass list; Use Retention Time = True; RT Tolerance [min] = 0.15; Mass Tolerance = 5ppm |
| Map to Metabolika Pathways | Search Settings | Metabolika Pathways = all included in CD 3.3.0.550; Search Mode = By Formula or Mass |
|  | By Mass Search Settings | Mass Tolerance = 5 ppm |
|  | By Formula Search Settings | Maximum Number of Predicted Compositions to be searched = 3 |
|  | Display Settings | Maximum Number of Pathways in ‘Pathways’ column = 20 |
| Predict Compositions | Prediction Settings | Mass Tolerance = 5 ppm; Minimum Element Counts = C,H; Maximum Element Counts = C90 H190 Br3 Cl4 K2 N10 Na2 O18 P3 S5; Minimum RDBE = 0; Maximum RDBE = 40, Minimum H/C = 0.1, Maximum H/C = 4; Maximum Number of Candidates = 10; Maximum Number of Internal Candidates = 200 |
|  | Pattern Matching | Intensity Tolerance [%] = 30; Intensity Threshold [%] = 0.1; S/N Threshold = 3; Minimum Spectral Fit [%] =30; Minimum Pattern Coverage [%] = 90; Use Dynamic recalibration = True |
|  | Fragments Matching | Use Fragments Matching = True; Mass Tolerance = 5 ppm; S/N Threshold = 3 |
| Assign Compound Annotations | General Settings | Mass Tolerance = 5ppm |
|  | Data Sources | Data Source #1 = MassList Search; Data Source #2 = mzVault Search; Data Source #3 = Predicted Compositions |
|  | Scoring Rules | Use mzLogic = True; Use Spectral Distance = True; SFit Threshold = 20; SFit Range =20 |
|  | Reprocessing | Clear Names = False |
| Mark Background Compounds | General Settings | Maximum Sample/Blank = 3; Maximum Blank/Sample = 0; Hide Background = True |

**3.2** **Data Quality and Data Filtering**

**Signal Response Evaluation**

We evaluated the signal response of the pooled sample with the intention of evaluating the fundamental principle of LC-MS/MS that if more of a given feature is present, a corresponding increase in the signal should be obtained. To do this, the pooled QC sample was injected and analyzed at three volumes in technical triplicate: 2 µL, 4 µL, and 6 µL, where 4 µL is the amount of material injected during the untargeted metabolomics assay in the present experiment. For any given feature, the peak area of the feature (*i.e.* the integration of a signal of unique *m/z* over a specific retention time) in the 4 µL sample is assumed to represent the mean value of that feature in the dataset; the peak area of that feature in the 2 µL sample and 6 µL sample should therefore represent 50% and 150% of the mean value, respectively. Multiple metrics were used to evaluate the signal response: Spearman’s p (evaluates a monotonic response), Pearson’s r (evaluates a linear response), and coefficient of determination (R^2^, evaluates fit to a linear model); for Spearman and Pearson correlations, p=0.05 was used as the statistical metric for significance. For every feature in the dataset, a value of these metrics was calculated to evaluate the signal response for that feature over the QC range. Any feature for which the value does not meet the filtering parameter, or any feature with a negative correlation (Spearman or Pearson), is filtered out of the dataset, ensuring that only features displaying positive correlations within the p=0.05 parameter are retained for further evaluation and interpretation.

Reference:

Overdahl, K.E., Collier, J.B., Jetten, A.M., Jarmusch, A.K. (2023). Signal Response Evaluation Applied to Untargeted Mass Spectrometry Data to Improve Data Interpretability. *Journal of the American Society for Mass Spectrometry*. DOI:10.1021/jasms.3c00220

**Assessment of Dispersion Ratio**

The pooled QC was utilized to calculate the dispersion ratio for the sample set. The dispersion ratio is a metric for describing the measurement precision of a detected metabolite; the metric focuses on statistical dispersion of the pooled QC samples in relation to the dispersion of the biological test samples. If the distribution of both the biological test sample measurements and the QC random error are Gaussian, then the dispersion ratio (D-ratio) is the ratio of the sample standard deviation for the pooled QC samples to the sample standard deviation for the biological test samples. (If the data distribution is not Gaussian, then the raw data must be mathematically transformed.) D-ratio is a measurement of technical variance: a D-ratio of 0% means that the technical variance is zero (a “perfect” measurement), and all observed variance can therefore be attributed to a non-measurement (putatively biological) cause, whereas a D-ratio of 100% indicates that all variance can be attributed to the measurement.

Reference:

Broadhurst, D., Goodacre, R., Reinke, S.N., Kuligowski, J., Wilson, I.D., Lewis, M.R., Dunn, W.B. (2018). Guidelines and considerations for the use of system suitability and quality control samples in mass spectrometry assays applied in untargeted clinical metabolomic studies. *Metabolomics* 14:72. DOI:10.1007/s11306-018-1367-3

**3.3** **Annotation**

Features were annotated based on MS/MS spectral matching, in alignment with the Metabolomics Standards Initiative guidelines (Level 2). Prior to annotation, the data acquisition strategy AcquireX (Thermo Fisher Scientific) was used to improve coverage compared to similar data acquisition strategies (*i.e.* data-dependent acquisition). The public, commercial, and in-house MS/MS spectral libraries NIST2020, GNPS (accessed 04-01-2022), mzCloud (offline, endogenous metabolites) were used to evaluate MS/MS spectral matches, as well as an in-house MS/MS spectral library acquired from authentic chemical standards purchased and analyzed by the MCF. The MS/MS spectral matching was performed in Compound Discoverer (Thermo Fisher Scientific).

During processing in the Jupyter Notebook, each feature was assigned an MSI level of confidence based on the measured data (without manual review). MSI Level 2 features have an MS/MS match to either a database entry or an in-house library reference match. MSI Level 4 and Level 5 features have either an MS/MS match but no database match, or no MS/MS match. MSI Level 2 features were promoted to MSI Level 1 after matching in-house *m/z* and retention time lists acquired on the same analytical platform using identical analytical conditions. The MSI Level 1 annotations are referred to as identified. Where appropriate, select annotations of statistical significance were confirmed with analytical standards (*i.e.* itaconic acid, citraconic acid, and mesaconic acid) and analyzed via the identical LC-MS method at a concentration of 1 µg mL^-1^.

**Reference**

Fiehn, O., Robertson, D., Griffin, J., van der Werf, M., Nikolau, B., Morrison, N., Sumner, L.W., Goodacre, R., Hardy, N.W., Taylor, C., Fostel, J., Kristal, B., Kaddurah-Daouk, R., Mendes, P., van Ommen, B., Lindon, J.C., Sansone, S.A. (2007). The metabolomics standards initiative (MSI). *Metabolomics* 3: 175–178. DOI:10.1007/s11306-007-0070-6.
